# Moderate chlorophyll-*a* environments reduce coral bleaching during thermal stress in Yap, Micronesia

**DOI:** 10.1038/s41598-023-36355-2

**Published:** 2023-06-08

**Authors:** Rachael Keighan, Robert van Woesik, Anthony Yalon, Joe Nam, Peter Houk

**Affiliations:** 1 University of Guam Marine Laboratory, UoG Station, Mangilao, GU 96923 USA; 2 Institute for Global Ecology, Florida Institute of Technology, 150 West University Blvd, Melbourne, Fl 32901 USA; 3 Yap State Division of Marine Resources, Colonia, Yap FM 96943 Federated States of Micronesia; 4 Yap Community Action Program, Colonia, Yap FM 96943 Federated States of Micronesia

**Keywords:** Climate-change ecology, Marine biology

## Abstract

Thermal-stress events on coral reefs lead to coral bleaching, mortality, and changes in species composition. The coral reefs of Yap, in the Federated States of Micronesia, however, remained largely unaffected by major thermal-stress events until 2020, when temperatures were elevated for three months. Twenty-nine study sites were examined around Yap to determine geographical and taxonomic patterns of coral abundance, bleaching susceptibility, and environmental predictors of bleaching susceptibility. Island-wide, 21% (± 14%) of the coral cover was bleached in 2020. Although inner reefs had a greater proportion of thermally-tolerant *Porites* corals, the prevalence of bleaching was consistently lower on inner reefs (10%) than on outer reefs (31%) for all coral taxa. Corals on both inner and outer reefs along the southwestern coast exhibited the lowest prevalence of coral bleaching and had consistently elevated chlorophyll-*a* concentrations. More broadly, we revealed a negative relationship between bleaching prevalence and (moderate) chlorophyll-*a* concentrations that may have facilitated resistance to thermal stress by reducing irradiance and providing a heterotrophic energy source to benefit some corals exposed to autotrophic stress. Southwestern reefs also supported a high but declining fish biomass, making these bleaching-resistant and productive reefs a potential climate-change refuge and a prime target for conservation.

## Introduction

The past three decades of climate change have seen a rise in the intensity of thermal-stress events, raising concerns about the future of coral reefs^1,2^. Thermal-stress events disrupt the relationship between host corals and their symbiotic microalgae, leading to coral-host starvation and mass mortality of corals when bleached^3^. Global studies have documented extensive coral loss from thermal-stress events^4,5^, with consistent susceptibility patterns across coral taxa^6–8^. In the past, widespread coral bleaching was only associated with El Niño-Southern Oscillation (ENSO) events, but coral bleaching is now associated with many climate-change-related oceanographic and climate features beyond ENSO. As a consequence, modern coral reefs are experiencing less time to recover between thermal-stress events than in the past^8–10^. To potentially mitigate these frequent bleaching events, management must identify (i) local factors that influence bleaching susceptibility and (ii) local and regional stressors that influence subsequent recovery.

Some environmental factors, such as turbidity, have been documented to increase bleaching resistance over regional scales^11^; however, relatively few studies have examined the spatial distribution of bleaching resistance at local scales^12–14^. One such study following the 2010 ENSO event in Palau noted that coral bleaching was lowest on nearshore reefs where reduced irradiance, through moderate turbidity, appeared to enhance thermal tolerance^13^. Yet, studies in the Florida Keys^15^, the Great Barrier Reef^16^, and French Polynesia^17^ showed higher bleaching where nutrient concentrations were elevated. Corals in controlled field and laboratory experiments also showed both positive^18,19^ and negative^20–22^ effects of dissolved inorganic nitrogen on bleaching susceptibility that may be a consequence of moderate versus high concentrations, respectively. In addition, corals in Mauritius experienced more intense bleaching on the windward coast where water flow rates were higher and temperature fluctuations were less variable than on the leeward coast^12^. Clearly, there are interactions among temperature, light, nutrients, and water-flow rates that influence bleaching susceptibility. These interactions need further investigation across a suite of spatial scales, especially at local scales where environmental factors are most dynamic, while also accounting for taxonomic variations in bleaching susceptibility.

Here, we examined bleaching susceptibility in response to the first major thermal-stress event in Yap, Micronesia. We used a stratified sampling approach to identify spatial differences in the bleaching of different coral genera across reef types and geography. In addition, we used satellite-derived data on chlorophyll-*a* concentrations and temperature to determine whether these environmental variables influenced coral bleaching during thermal stress. The differential susceptibilty of coral taxa to heat stress we observed mainly followed past studies; however, novel relationships were revealed between moderate chlorophyll-a environments and bleaching that will provide decision support for management strategies to mitigate the effects of climate change.

## Methods

### Study location and field methods

The main island of Yap is the largest of the 15 islands comprising Yap State (~ 100 km^2^) and is home to roughly two-thirds of the state’s 11,377 residents (FSM Statistics Office, 2020). Management of Yap’s marine resources is unique, balancing modern state-driven legislation with traditional reef tenure^23^. A strong sense of community involvement has been associated with the success of several marine protected areas (MPA) around the island^24^. Nonetheless, in recent years growing fishing pressure, intermittent Crown-of-Thorns seastar outbreaks, and to a lesser extent land-based pollution have caused declines in Yap’s coral-reef resources^25^.

**Figure 1:**
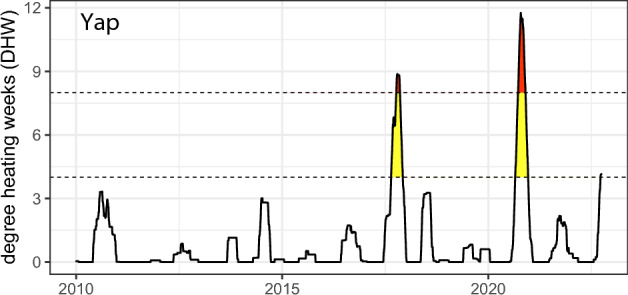
Degree heating weeks (°C) for Yap State, 2010 to 2022. Dashed lines indicate the thresholds of 4 °C weeks (yellow), thought to induce bleaching, and 8 °C weeks (red), associated with widespread bleaching mortality.

In 2017, Yap experienced its first heat-stress event when the Degree Heating Weeks (DHW) exceeded 8 °C-weeks during October (Fig. 1). Mild bleaching was observed for *Acropora* corals and a slight shift in dominance to *Porites* corals occurred over the next two years^26^. However, in the summer of 2020, the combination of a La Niña and a negative Pacific Decadal Oscillation, not associated with any ENSO cycle, led to prolonged thermal stress in the western Pacific Ocean. During this time, the 8 °C-weeks threshold^27^ was exceeded for nearly three months (Fig. 1). Previous research suggested that these oceanographic conditions may lead to significant heat stress for Yap^26^ and, coupled with National Oceanic and Atmospheric Administration (NOAA) Coral Reef Watch products at short time intervals, these predictions enabled local teams to secure the financial, logistical, and technical support needed to conduct island-wide surveys during the 2020 thermal-stress event.

**Figure 2:**
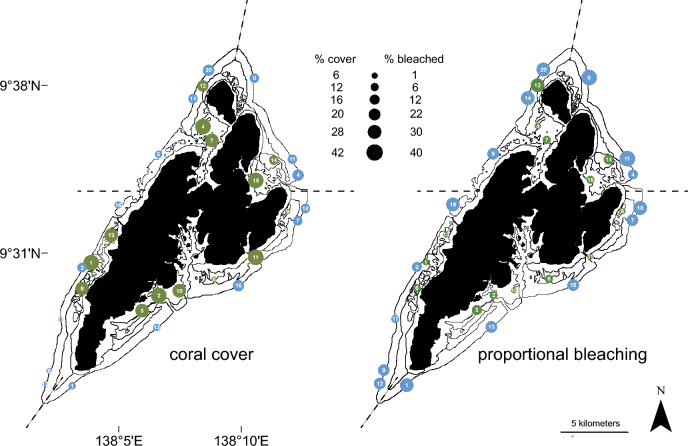
Coral cover (left) and proportional bleaching (right) across study sites. Circle size is proportional to the contribution of each variable. Outer and inner reef types are differentiated by blue and green circles, respectively. Numbers indicate unique site identifiers (Supplementary Table S1. Dashed lines indicate boundaries of the four geographic quadrants.

We used a stratified random survey design, with stratification across both inner and outer reefs and the four geographic sectors (northeast, northwest, southeast, and southwest). Overall, we surveyed 14 inner and 15 outer reefs distributed across Yap (Fig. 2). Snorkel surveys were conducted between November and December of 2020 following the thermal peak. At each site, three 30-m transect tapes were placed parallel to the 3-m depth contour. Along each transect, surveyors took a series of 1-m^2^ photo-quadrats, captured at 1-m intervals along the transect lines. These transects provided a total survey area of 90 m^2^ per site.

### Image analysis

Within each photo quadrat, corals were identified to genera following the taxonomy outlined by Veron^28^, Budd and Bosellini^29^, and Huang et al.^30^. The number of coral colonies within each genus was counted in each image. Estimates of total coral cover for each genus were determined to the nearest percentage by overlaying the images with a 10 cm by 10 cm grid. The prevalence of coral bleaching was similarly estimated to determine the overall proportion of coral colonies that were bleached. Using these methods, we evaluated a total of 29,221 coral colonies, ranging between 402 to 1611 colonies per site.

### Environmental variables

Environmental data were gathered to characterize the bleaching event and examine potential relationships between bleaching prevalence, sea-surface temperatures, and chlorophyll-*a* concentrations across Yap. Firstly, to evaluate the cumulative heat stress Yap had experienced since 2010, DHW data were accessed from the NOAA National Environmental Satellite, Data, and Information Services (NESDIS) that are served through the Pacific Islands Ocean Observing System (https://pae-paha.pacioos.hawaii.edu/erddap/index.html). We summarized DHW with respect to two established thresholds, representing bleaching warning and expected bleaching events (4 °C-weeks and 8 °C-weeks, respectively)^27^.

Additionally, sea-surface temperature and chlorophyll-*a* satellite data were downloaded from the NOAA Coastwatch server AVHRR Pathfinder and VIIRS data products, respectively (https://coastwatch.pfeg.noaa.gov/) to examine localized differences in these two potential drivers of spatial differences in bleaching susceptibility^8,11,16,31^. Site-level chlorophyll-*a* concentrations were derived for outer reefs from our satellite data by averaging the nearest six-cell values, and sea surface temperatures from the nearest two-cell values due to lower resolution. Shallow reef pixels were excluded from satellite-derived data because reef-associated pixels provide false estimates of chlorophyll-*a* and temperature depending on the size and shape of the reefs.

Finally, given a previous study which partitioned the contribution of different stressors, including fishing pressure and pollution, on Yap’s reefs and found that fishing pressure was a primary determinant of reef condition^32^, we accessed data on fish biomass from the long-term Micronesia Reef Monitoring database as an additional metric of human impact (https://micronesiareefmonitoring.com/). Fish data were collected using replicate, stationary-point-count surveys along five 50-m transects across 20 sites, stratified by reef type and MPA status^25,32^. While species-level data were collected during long-term monitoring, the fish-biomass data used in the present study were binned into six major guilds: (i) small herbivores/detritivores, (ii) large herbivores/detritivores, (iii) *Bolbometopon muricatum*, (iv) small secondary consumers, (v) large secondary consumers, and (vi) tertiary consumers (defined further by Houk et al*.*^33^). Temporal trends in fish biomass were graphically highlighted for outer reefs where data were collected near the present study sites used in the bleaching assessment.

### Data analysis

Coral cover and bleaching prevalence were visually compared by aggregating data at the site level and plotting proportional circles for both metrics on a map of the island. To compare bleaching prevalence across geographical sectors and reef types, while taking taxonomic differences in bleaching susceptibility into account, we used a linear mixed-effects model approach using the R package *lme4*^34^. Random effects were coral genera and transects within sites. Data on bleaching prevalence were used for each genus so that we did not violate any assumptions of independence when comparing across genera. Post-hoc comparisons were conducted when significant overall variation was detected across the geographic sectors, or between reef types. Both additive and interactive models were tested for the best fit, which was determined by Chi-Square examinations of the residuals and the Akaike Information Criterion (AIC). We also used standard least-squares regression models to examine the relationships between coral cover and bleaching prevalence within each coral genus. Additionally, to visualize spatial and taxonomic trends in bleaching, we created a heatmap organized by geography and reef type, using hierarchical clustering based on Bray–Curtis dissimilarities.

Given the observed spatial patterns in bleaching prevalence, we investigated potential associations between sea-surface temperature, chlorophyll-*a*, and bleaching prevalence for Yap’s outer reefs, where satellite observations were not influenced by shallow reef pixels. Linear mixed-effects models were used to compare bleaching prevalence against chlorophyll-*a*, sea-surface temperature, and their potential interaction. Models with both random slopes and intercepts for coral genera were investigated to account for potential differences in susceptibility to bleaching and magnitude of influence with chlorophyll-*a*. Sea-surface temperature data did not significantly improve model fit and were therefore excluded from our final model. All analyses were undertaken in R version 4.2.1^35^.

## Results

The first widespread coral bleaching event in Yap started in August 2020 and extended until November 2020, when the 8 °C-weeks threshold was surpassed for nearly three months (Fig. 1). Island-wide, 21% (*SD* = 14%) of coral cover was affected by bleaching; however, all reefs were not impacted equally. While inner reefs supported higher overall coral cover (*M* = 41%, *SD* = 22%) than outer reefs (*M* = 18%, *SD* = 4%), only 10% (*SD* = 9%) of the coral cover on inner reefs bleached, whereas 31% (*SD* = 9%) of the coral cover on outer reefs bleached (Fig. 2). Bleaching patterns also varied depending on coral genera, reef type, and geography.

**Figure 3:**
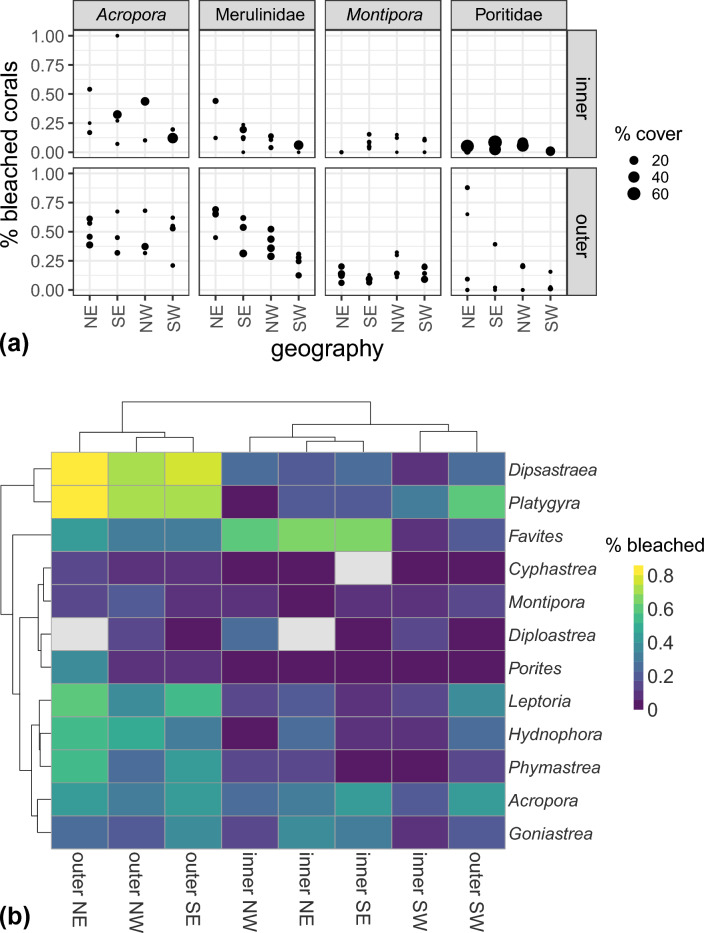
Trends in (**a**) coral cover and bleaching intensity for major compositional taxa across reef type and geographic quadrant. Circles represent individual sites within each geographic quadrant, and the size of each circle is proportional to coral cover at the corresponding site. (**b**) Similarities in bleaching response were grouped by geographical quadrant/reef type and coral genera. Dendrograms depict similarities in bleaching responses across coral genera, colors indicate proportional bleaching.

Corals in the genera *Acropora, Montipora,* and *Porites,* along with many genera within the family Merulinidae, were dominant on Yap’s reefs (Fig. 3, Supplementary Fig. S1). *Porites* dominated inner reefs whereas a more even coverage of all genera existed on outer reefs (Fig. 3a). Despite differences in coral community composition between reef types, linear mixed-effects models revealed that the prevalence of coral bleaching was significantly lower on inner reefs than on outer reefs for all genera (Table 1). Interestingly, geographic sector was also a significant predictor of bleaching prevalence for both inner and outer reefs. Bleaching was highest on northeastern reefs and lowest on southwestern reefs across all coral taxa (Table 1).

**Table 1:** Estimated coefficients and standard errors for the Linear Mixed Effects models for coral bleaching in Yap, Micronesia, in 2020.

	Bleaching prevalence model	Chlorophyll-a/bleaching resistance model
	Dependent variable	
	Proportion bleached	Proportion bleached (outer reefs)
NW	− 0.102** (0.032)	
SE	− 0.081* (0.033)	
SW	− 0.178*** (0.033)	
Reef type—outer	0.175*** (0.024)	
Chlorophyll-*a*		− 7.197*** (2.263)
Constant	0.255*** (0.051)	0.820*** (0.192)
Observations	269	169
Log likelihood	43.718	40.244
Akaike Inf. crit	− 73.437	− 68.487
Bayesian Inf. crit	− 48.274	− 49.708

Asterisks indicate the degree of significance.

*p < 0.05; **p < 0.01; ***p < 0.001.

**Figure 4:**
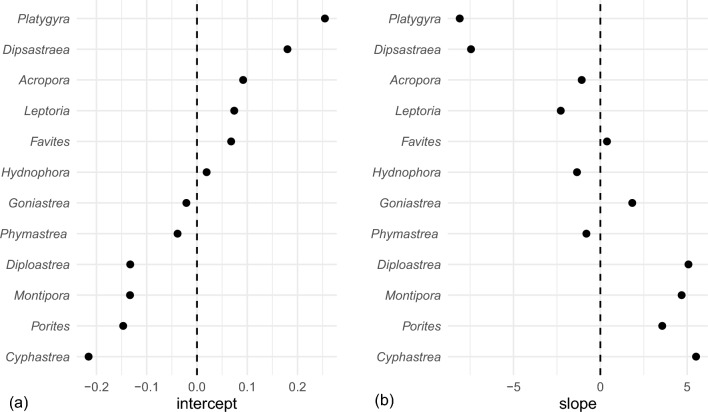
Random effects associated with linear mixed effects modeling. Random intercepts (**a**) of the bleaching-prevalence model highlight which coral genera were most sensitive or resistant to heat stress. Random slopes (**b**) of the chlorophyll-*a-*bleaching-resistance model indicate the relative magnitude of the relationship between bleaching prevalence and chlorophyll-*a* for each coral genus.

Significant random effects indicated which genera were most vulnerable to the thermal-stress event (Fig. 4a). Among the dominant genera, *Acropora* (*M* = 36%, *SD* = 17%), *Platygyra* (*M* = 56%, *SD* = 30%), *Dipsastraea* (*M* = 47%, *SD* = 31%), *Leptoria* (*M* = 38%, *SD* = 25%), *Favites* (*M* = 37%, *SD* = 23%), and *Hydnophora* (*M* = 30%, *SD* = 23%) were most sensitive to bleaching. By contrast, the most resistant corals were *Porites* (*M* = 10%, *SD* = 19%), *Cyphastrea* (*M* = 7%, *SD* = 10%), *Diploastrea* (*M* = 10%, *SD* = 17%), and *Montipora* (*M* = 12%, *SD* = 8%). No significant relationships were observed between coral cover and bleaching prevalence for any dominant genera. Thus, while coral composition and cover differed around Yap, these factors were either controlled for as random effects or were non-significant predictors of bleaching prevalence. Therefore, consistent patterns of bleaching prevalence that existed across reef types and geography, for all coral taxa, appeared to be related to different environmental settings.

Cluster analyses revealed similar patterns in bleaching susceptibility to those described above for the major taxa across the geographic quadrants and reef types (Fig. 3b). For reefs in the northeastern, northwestern, and southeastern sectors, patterns in bleaching appeared to be most strongly influenced by reef type, with outer reefs experiencing the highest bleaching prevalence. However, the southwestern reefs showed a high affinity and separation in the cluster analysis, suggesting a different environmental setting than elsewhere in Yap.

**Figure 5:**
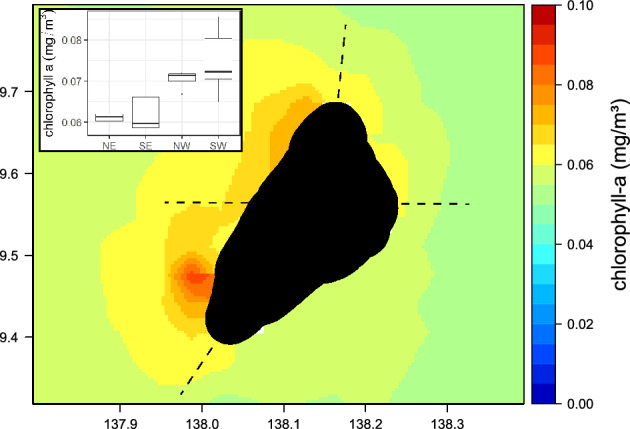
Ten-year long-term chlorophyll-*a* data between 2010 and 2020 (mg m^−3^), obtained from NOAA VIIRS server (https://coastwatch.pfeg.noaa.gov/), for Yap. Raster data were interpolated to create a smooth vector surface. Boxplots indicate chlorophyll-*a* concentrations at outer reef sites within each of the geographic quadrants (*NE* northeast, *SE* southeast, *NW* northwest, *SW* southwest).

While satellite data indicated that sea-surface temperatures were relatively uniform around the island during the bleaching event, and over the past 10 years (Supplementary Fig. S2), 10-year primary productivity was more spatially variable. Higher concentrations of chlorophyll-*a* were observed along the leeward, west coast than along with the exposed, east coast, and a distinct hotspot was noted in the southwestern quadrant (Fig. 5). Linear mixed effects model revealed that chlorophyll-*a* significantly reduced bleaching prevalence for all genera on outer reefs except *Montipora* and *Diploastrea* (Table 1, Supplementary Fig. S3). Interestingly, the magnitude (i.e. slope) of this relationship was greatest for coral genera with the greatest bleaching susceptibility (Fig. 4).

**Figure 6:**
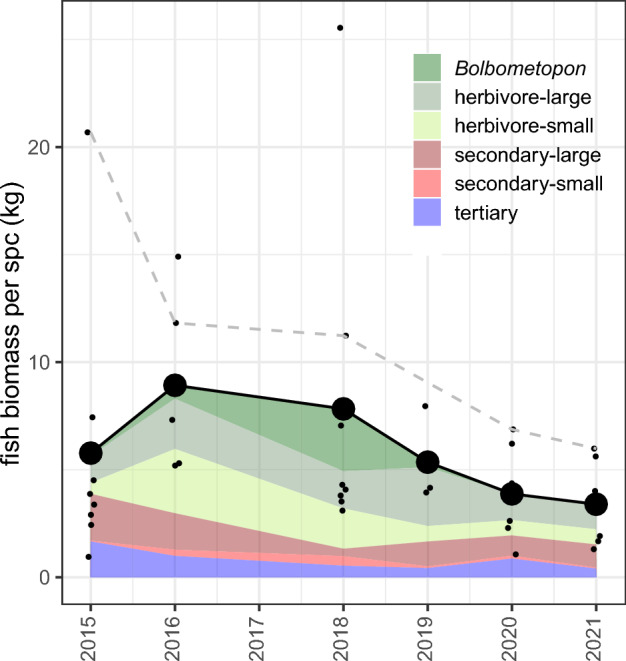
Trends in fish biomass from replicate stationary-point-count (SPC) surveys for Yap. Colors indicate fish guilds. Small black dots indicate individual reefs, whereas large black dots indicate annual means. Dashed line indicates fish biomass at one long-term site associated with the southwestern quadrant where enhanced productivity was observed.

Additionally, we found that southwestern reefs, where primary productivity was greatest, also consistently supported the highest fish biomass around the island, exceeding island-wide averages by approximately 30% (Fig. 6).

## Discussion

Yap was one of the few Pacific islands to escape major thermal stress and coral bleaching over the past three decades. The island’s first major coral bleaching event occurred in the late summer of 2020 when unprecedented heat stress caused 21% of shallow-water corals to bleach. However, there was significant variability among coral genera, across reef types, and among geographic quadrants. Taxonomic trends generally followed studies in other parts of the Indo-Pacific, with thermally-tolerant genera including *Cyphastrea, Diploastrea, and Porites* experiencing less bleaching than thermally-sensitive genera, such as *Acropora*^36–38^*.* Yet, *Montipora,* which is reported among the most sensitive corals^36,37^, experienced comparatively low bleaching in Yap. Conversely, *Dipsastraea* (formerly *Favia*) and *Platygyra*, which are reported among the more resilient corals^7,38^, experienced the most severe bleaching across all reef locations. Our findings reinforce that, while general taxonomic trends tend to be well established for dominant corals such as *Porites* and *Acropora*, there are differences in coral bleaching susceptibility across biogeographic regions.

At the local scale, the present study showed more extensive bleaching on outer reefs than inner. The high bleaching resistance of Yap’s inner reefs was similar to reports from nearby Palau, where corals on reefs in sheltered bays exhibited lower bleaching prevalence during a 2010 thermal-stress event^13^. Greater resistance to bleaching in Palau’s bays was attributed to moderate turbidity which reduced irradiance. These findings echoed observations from Bermuda, where bleaching was lowest for coral colonies on inshore reefs^39^. Similarly, on the Great Barrier Reef, Morgan and colleagues^40^ showed lower bleaching on turbid reefs than elsewhere, despite high thermal stress. Our findings supported the conclusions of these studies and suggested that the stress-tolerant coral communities and turbid conditions of inner reefs make them potential refugia against climate change. However, we add that moderate productivity may also contribute to bleaching resistance and may be difficult to disentangle from turbidity.

Despite little temperature variability around the island, we showed differences in the prevalence of bleaching across Yap’s four geographic quadrants. Windward reefs in the northeast were most heavily impacted by thermal stress and leeward reefs in the southwest were most resistant. McClanahan and colleagues^12^ described similar geographic trends around the island of Mauritius and concluded that higher water flow on windward reefs reduced background stressors and stabilized environmental conditions, leading to the establishment of less thermally-tolerant coral communities. Alternatively, we suggest that productivity contributed to our spatial trends as coral bleaching resistance was greatest where chlorophyll-*a* concentrations were moderately elevated.

The accumulation of chlorophyll-*a* along the leeward coast was attributed to the ‘island mass effect’, whereby oceanographic eddies form in the wake of islands which enhance productivity^41,42^. Variation in nearshore productivity has been linked to trophic ecology with one model coral, *Pocillopora meandrina*, acquiring a greater proportion of its nutrition heterotrophically in regions of high primary productivity^43^. While not applicable to all species, similar variations in heterotrophy may have contributed to the observed resistance to bleaching among productive reefs^44^. In support, Hughes et al*.* (Extended Data Table 1)^8^ showed that chlorophyll-*a* reduced the impact of thermal stress on corals across the Great Barrier Reef based upon concentrations that were approximately one order of magnitude greater than on the southwest reefs of Yap. We synthesize that the moderate productivity found on inner and southwestern reefs may have induced bleaching resistance by (i) providing a supplemental food source for corals able to utilize a heterotrophic diet during periods of autotrophic stress, and (ii) shading corals from the interaction between high temperatures and high irradiance during the bleaching event. While further work is required to appreciate the influences of nutrients, shading, primary production on bleaching resistance, our findings suggest that the most susceptible corals to thermal and irradiance stress experienced the greatest benefits most from these two mechanisms.

Our findings of increased bleaching resistance of corals where chlorophyll-*a* was moderate add to the rapidly increasing literature involving coral bleaching susceptibility in response to thermal stress, chlorophyll-*a*, and nutrient concentrations^15,17,19–21,45–48^. Given the contrasting reports of coral bleaching intensity at differing levels of productivity, in combination, these studies suggest that a hump-shaped relationship may exist between productivity in the water column and coral-bleaching resistance. Increasing productivity in the water column increases bleaching resistance up to a threshold, beyond which high productivity may become detrimental for corals. In support, coral growth has long been known to exhibit a humped relationship with suspended organic matter^49^ and a similar relationship between nutrients and species diversity drives the classic ‘paradox of enrichment’^50^. Yet, moderate productivity likely acts in concert with other environmental regimes to determine bleaching resistance across local scales.

The inverse relationship between sensitivity to thermal stress in and out of moderately productive areas (Fig. 4) suggests that areas of moderate productivity, such as those found in Yap’s southwest, may act as refugia for otherwise thermally sensitive coral populations. In order to preserve these thermally resistant southwestern reefs, additional protection from local fishing pressure and land-based pollution may be warranted. Unfortunately, long-term fisheries monitoring data for Yap suggested that the high-fish biomass once evident in southwestern reefs is now declining faster than on other reefs. Furthermore, previous studies have shown that fishing pressure is highest among inner reefs, close to humans, and with low wind-wave exposure^24^. Using MPAs as one management tool, our results can help guide MPA placement, timing, and duration. For example, the implementation of temporary protection during and after heat stress may be warranted for exposed northern and eastern reefs, where the greatest coral impacts were observed. Temporary MPAs, for 3–4 years, would maximize the beneficial responses of fish assemblages following bleaching, as observed across Micronesia^33^. By contrast, long-term fisheries management efforts may be prioritized on leeward reefs that are easily accessed because of low wave energy, and are located near eddies that fuel fisheries production, larval retention, and anecdotal spawning aggregations.

In conclusion, our study revealed that the resistance of corals to an unprecedented thermal-stress event on Yap was highest among inner and southwestern reefs where moderate chlorophyll-*a* concentrations existed. While differences in thermal resistance across reef types have been well established^13,39^, our findings of resistance along Yap’s southwestern reefs, associated with oceanographic productivity, were novel but likely acted in concert with other factors including differences in irradiance associated with changes in productivity. Spatial patterns in bleaching resistance around Yap suggested that heat-stress mitigation strategies should be incorporated into evolving management strategies that deal with both local and global stressors.

## Supplementary Information

Supplementary Figures.

Supplementary Table S1.

Supplementary Table S2.

## Data Availability

Data are provided in Supplementary Tables S1 and S2.
