# Supplementary material for: Moderate chlorophyll-a environments reduce coral bleaching during thermal stress in Yap, Micronesia
Source: Sci Rep. 2023 Jun 8;13:9338. doi: 10.1038/s41598-023-36355-2 (PMC10250426; doi:10.1038/s41598-023-36355-2)
Supplement: Supplementary file 1 — Supplementary Figures. [file 41598_2023_36355_MOESM1_ESM.pdf]

Supplementary Figures

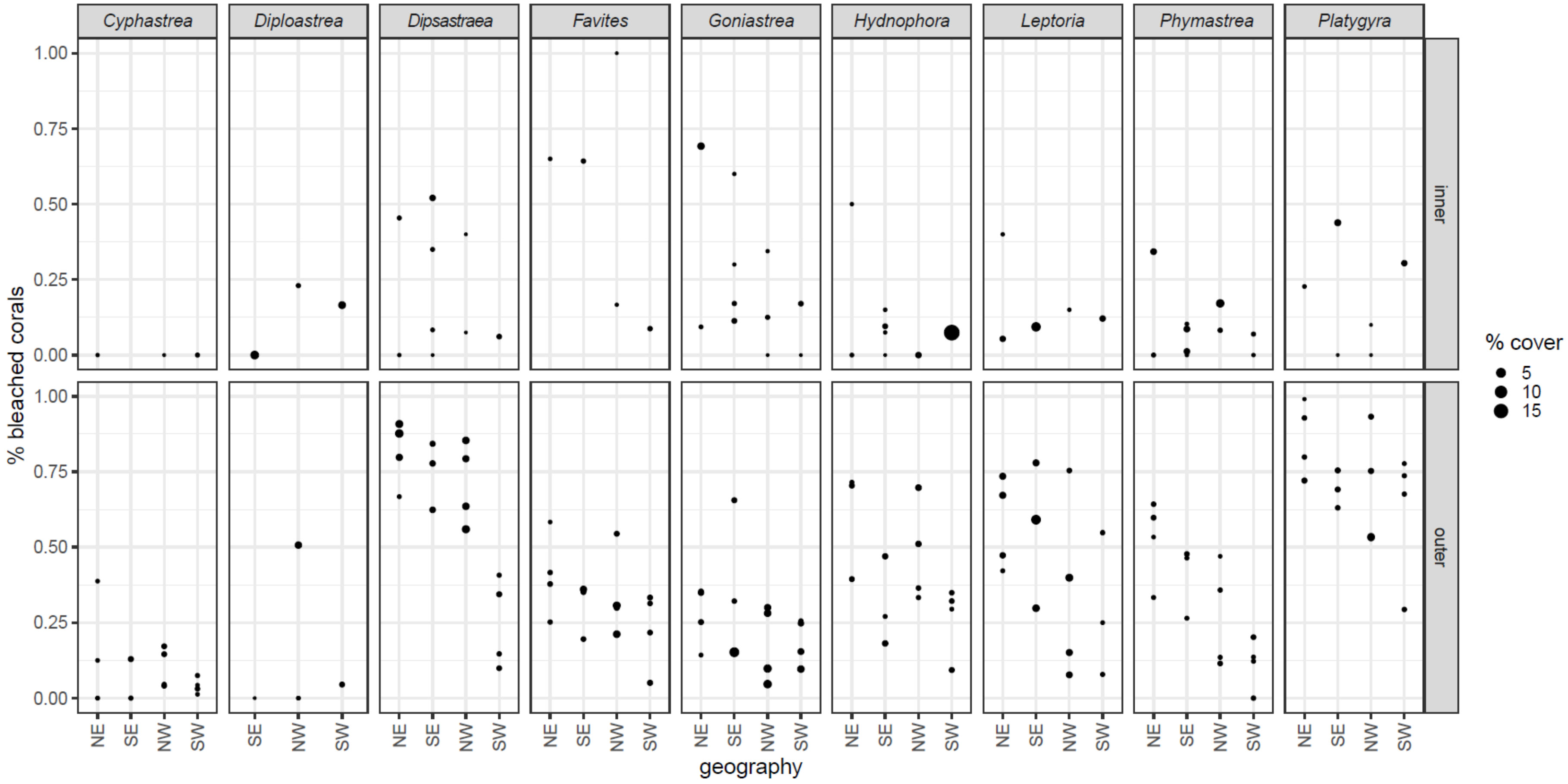

Supplementary Figure S1. Coral cover and proportional contribution for genera in the family Merulinidae included in taxonomic analyses. All genera exceeded 0.5% of total coral cover for inner and outer sites. Circles represent individual sites within geographic quadrants, and circle size represents coral cover at the corresponding site.

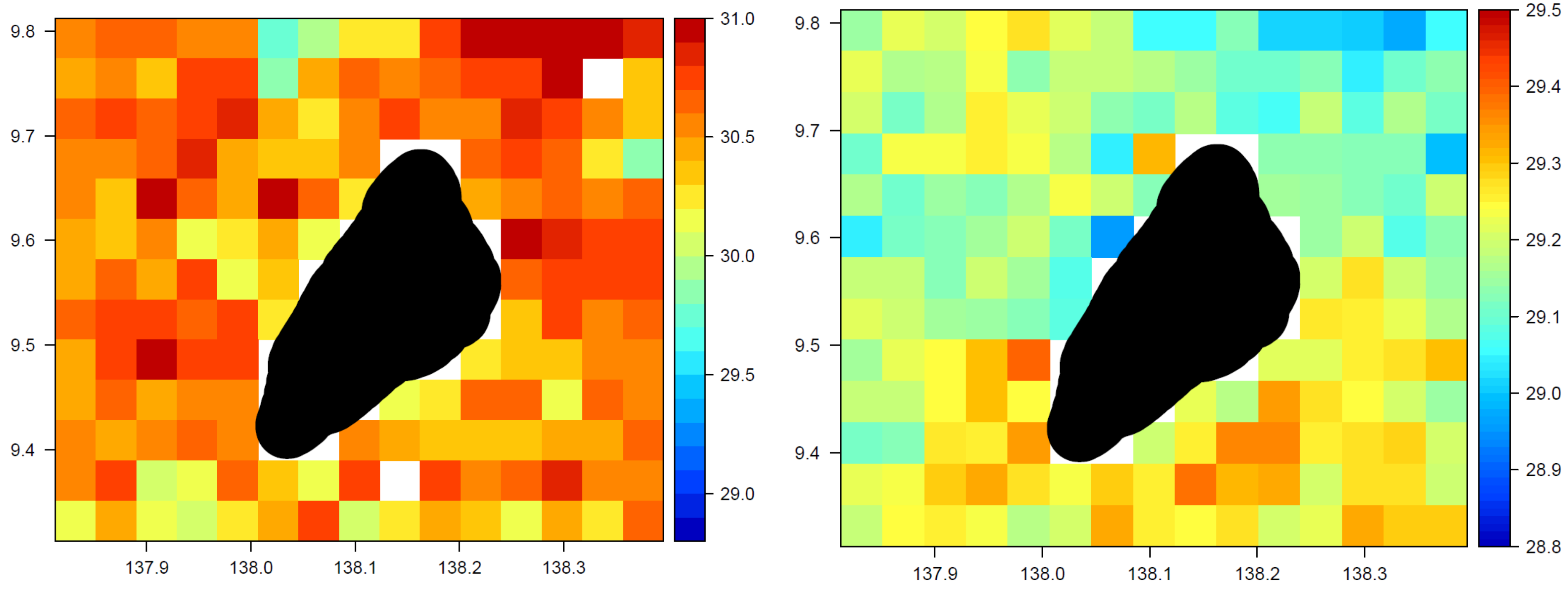

Supplementary Figure S2. Sea surface temperatures (°C) for Yap (a) during the 2020 thermal-stress event and (b) between 2010 and 2020, obtained from NOAA Pathfinder server (https://coastwatch.pfeg.noaa.gov/).

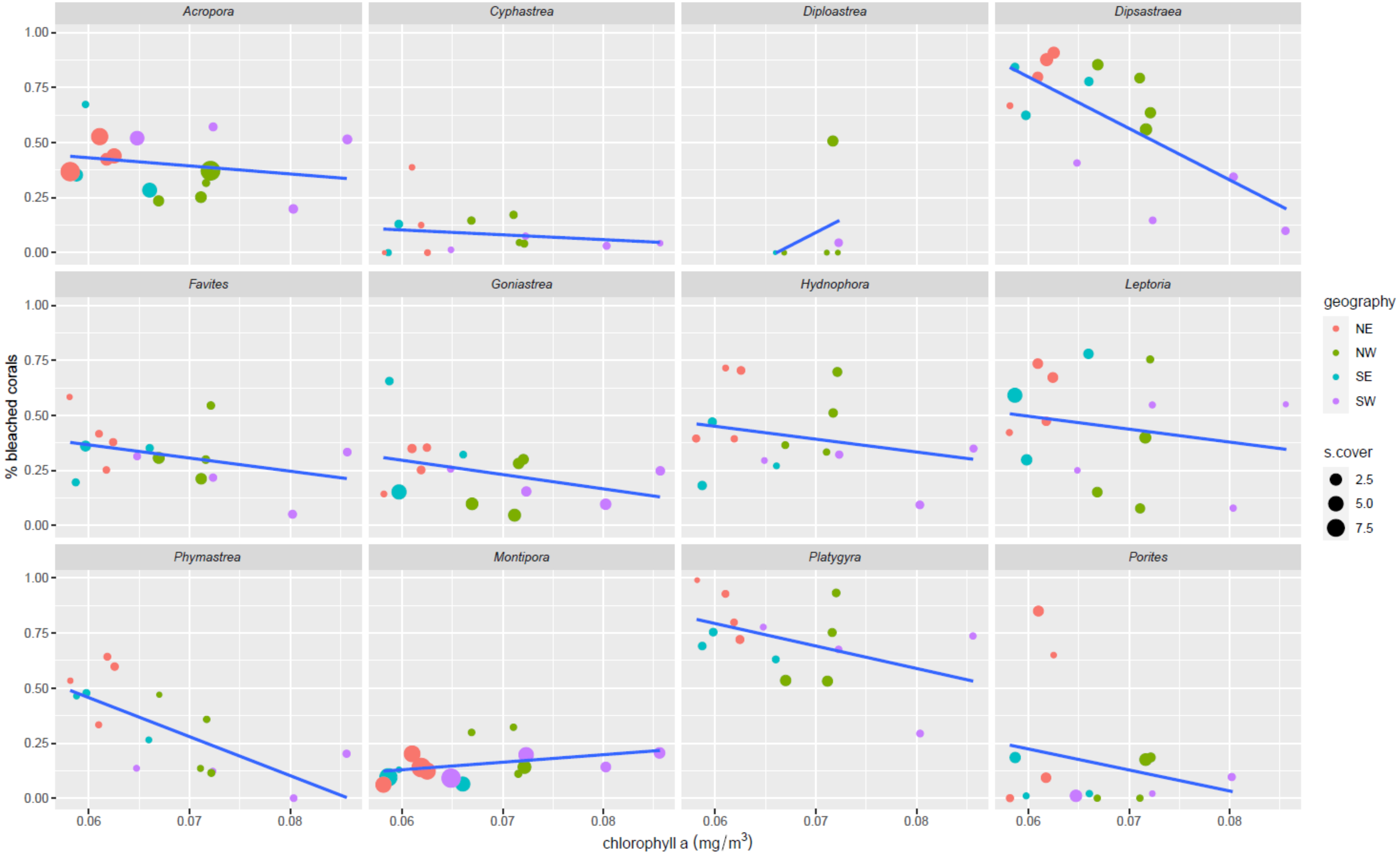

Supplementary Figure S3. Linear regressions of chlorophyll-*a* and proportion bleached for outer reef coral genera. Colored dots indicate geographic quadrants and circle size represents coral cover at each site.
