# Supplementary material for: Moderate chlorophyll-a environments reduce coral bleaching during thermal stress in Yap, Micronesia
Source: Sci Rep. 2023 Jun 8;13:9338. doi: 10.1038/s41598-023-36355-2 (PMC10250426; doi:10.1038/s41598-023-36355-2)
Supplement: Supplementary file 2 — Supplementary Table S1. [file 41598_2023_36355_MOESM2_ESM.pdf]

Proportional Bleaching

site	reeftype	side	geography	*Leptoria*	*Dipsastraea*	*Diploastrea*	*Hydnophora*	*Goniastrea*	*Favites*	*Phymastrea*	*Cyphastrea*	*Platygyra*	*Acropora*	*Montipora*	*Porites*
1	inner	W	SW	0.121	0.061111111	0.165454545	0.074253731	0.17025641	0.087419355	0.0695	0	0.304347826	0.330291262	0.116666667	0.007873404
1	outer	E	SE	0.779357798	0.77739726	0	0.270588235	0.321428571	0.351162791	0.264705882	0	0.630434783	0.283716814	0.064305949	0.020588235
2	inner	E	SE	0	0	0	0.15	0.6	0	0	0	0	0.27	0.071428571	0.084257674
2	outer	W	SW	0.25	0.407275192	0	0.294902549	0.255844466	0.313758493	0.13598355	0.012820513	0.776923077	0.520154281	0.091017391	0.010656455
3	inner	E	SE	0	0.35	0	0.075	0.1708	0	0.086111111	0	0	0.323689956	0.046794872	0.047118812
4	inner	W	NW	0	0.075	0	0	0.125	1	0.082142857	0	0	0.101785714	0.123076923	0.054183831
5	outer	W	NW	0.077130745	0.793040146	0	0.333127208	0.046453901	0.211854324	0.135135135	0.171666667	0.531625909	0.251918334	0.322202797	0
6	inner	W	SW	0	0	0	0	0	0	0	0	0	0.121296748	0.103424658	0.017295597
7	inner	W	NW	0	0.4	0.23	0	0	0	0.171261682	0	0	0	0	0.080875096
7	outer	E	NE	0.421761658	0.667484663	0	0.394649227	0.142772861	0.583333333	0.53362069	0	0.990517241	0.367812356	0.061150016	0
8	inner	E	SE	0	0.083333333	0	0	0.3	0	0.102857143	0	0	0.79	0.153764706	0.052930757
8	outer	E	NE	0.734954955	0.797560976	0	0.715	0.349104478	0.416129032	0.333333333	0.3875	0.928125	0.526986028	0.201413276	0.85
9	inner	E	NE	0	0	0	0	0	0	0	0	0	0.25	0	0.007017544
9	outer	W	SW	0.55	0.099047619	0	0.349142857	0.247808219	0.333125	0.201923077	0.042857143	0.736666667	0.514404762	0.205695364	0
10	inner	E	SE	0	0	0	0	0	0	0	0	0	0	0.032289951	0.024332969
11	inner	E	SE	0.093416928	0.520588235	0	0.095454545	0.11326087	0.642592593	0.012048193	0	0.438425926	0.2234375	0.0875	0.011636578
11	outer	E	NE	0.671982759	0.908146067	0	0.704	0.353409091	0.37804878	0.597727273	0	0.720689655	0.439458689	0.123239437	0.65
12	inner	W	NW	0.15	0	0	0	0.344217687	0.166666667	0	0	0.1	0.43533582	0.15	0.029335634
12	outer	W	SW	0.547222222	0.146551724	0.045	0.321538462	0.154285714	0.217073171	0.122222222	0.074782609	0.676	0.57122807	0.196763926	0.020833333
13	inner	W	SW	0	0	0	0	0	0	0	0	0	0.1	0	0.007726316
13	outer	E	SE	0.297876712	0.62375	0	0.469850746	0.152071823	0.360393701	0.477435897	0.129464286	0.754339623	0.673076923	0.129230769	0.010526316
14	inner	E	NE	0.053690685	0.45412844	0	0	0.692045117	0.65	0.34257885	0	0.227218935	0.168823147	0	0.029950229
14	outer	W	NW	0.399011628	0.559424084	0.507037037	0.510810811	0.28137931	0.299038462	0.358	0.045714286	0.752459016	0.316666667	0.110526316	0.175333333
15	inner	E	NE	0.4	0	0	0.5	0.093333333	0	0	0	0	0.540540541	0	0.055489675
15	outer	E	SE	0.591140603	0.842680262	0	0.181271091	0.655560065	0.195568928	0.463896104	0	0.691176471	0.352597403	0.094984359	0.184644412
16	outer	W	NW	0.151130435	0.853716216	0	0.364705882	0.098019324	0.30683908	0.47	0.145555556	0.534685315	0.235185185	0.298571429	0
17	outer	W	SW	0.078571429	0.344074074	0	0.093137255	0.09625	0.050701754	0	0.030882353	0.293666667	0.198648649	0.141666667	0.096153846
18	outer	E	NE	0.473055556	0.876711712	0	0.393333333	0.251923077	0.251923077	0.642424242	0.125	0.79862069	0.424251208	0.13921875	0.092957746
20	outer	W	NW	0.753947368	0.63556338	0	0.697159091	0.3	0.544680851	0.114864865	0.040789474	0.932352941	0.371883469	0.141385768	0.185
