# Supplementary material for: Moderate chlorophyll-a environments reduce coral bleaching during thermal stress in Yap, Micronesia
Source: Sci Rep. 2023 Jun 8;13:9338. doi: 10.1038/s41598-023-36355-2 (PMC10250426; doi:10.1038/s41598-023-36355-2)
Supplement: Supplementary file 3 — Supplementary Table S2. [file 41598_2023_36355_MOESM3_ESM.pdf]

% Coral Cover

site	reeftype	side	geography	*Leptoria*	*Dipsastraea*	*Diploastrea*	*Hydnophora*	*Goniastrea*	*Favites*	*Phymastrea*	*Cyphastrea*	*Platygyra*	*Acropora*	*Montipora*	*Porites*
1	inner	W	SW	0.44	0.173333333	0.08	3.64	0.373333333	0.16	0.266666667	0.4	0.12	0.733333333	0.173333333	9.133333333
1	outer	E	SE	1.253333333	0.746666667	0.04	0.2	0.3	0.293333333	0.213333333	0	0.34	2.84	4.413333333	0.12
2	inner	E	SE	0	0.04	0	0.08	0.12	0	0	0	0	0.08	0.093333333	10.22666667
2	outer	W	SW	0.182291667	0.389027778	0	0.195694444	0.259861111	0.47	0.185833333	0.123333333	0.197916667	1.626527778	7.013333333	0.497222222
3	inner	E	SE	0	0.26	0.04	0.2	0.466666667	0	1.773333333	0	0	5.813333333	1.12	5.306666667
4	inner	W	NW	0	0.04	0	0	0.56	0.04	0.493333333	0	0	0.34	0.293333333	14.68
5	outer	W	NW	1.074202899	1.83942029	0.043478261	0.232463768	2.409275362	1.095072464	0.257391304	0.58	0.877681159	1.227246377	0.235942029	0.112463768
6	inner	W	SW	0	0	0	0	0.08	0	0.16	0	0	5.84	1.093333333	0.853333333
7	inner	W	NW	0	0.04	0.14	0.16	0.12	0	4.16	0	0.04	0	0.04	5.826666667
7	outer	E	NE	0.121818182	0.239393939	0	0.506060606	0.232121212	0.167272727	0.129090909	0.04	0.055151515	1.663636364	4.258787879	0.24
8	inner	E	SE	0	0.14	0	0.04	0.1	0	0.12	0	0.04	0.2	0.893333333	4.08
8	outer	E	NE	1.133333333	1.133333333	0	0.266666667	1.066666667	0.293333333	0.226666667	0.24	0.306666667	2.986666667	5.36	0.88
9	inner	E	NE	0	0	0	0	0	0	0	0	0	0.24	0	4.853333333
9	outer	W	SW	0.1	0.6	0	0.373333333	1.533333333	0.76	0.74	0.42	0.32	1.16	3.053333333	0
10	inner	E	SE	0	0	0	0	0	0	0.04	0	0	0	0.27	8.195
11	inner	E	SE	0.666666667	0.3	0.14	0.373333333	0.253333333	0.2	0.6	0	0.253333333	0.68	0.34	7.36
11	outer	E	NE	1.413333333	1.706666667	0	0.546666667	0.533333333	0.36	0.413333333	0.22	0.453333333	1.133333333	4.333333333	0.08
12	inner	W	NW	0.041666667	0	0	0	0.082222222	0.04	0	0.083333333	0.04	7.288888889	0.041666667	0.433888889
12	outer	W	SW	0.24	0.4	0.24	0.666666667	0.973333333	0.586666667	0.24	0.253333333	0.346666667	0.84	6.386666667	0.066666667
13	inner	W	SW	0	0	0	0	0	0	0	0	0	0.08	0.04	12.09333333
13	outer	E	SE	2.573333333	1.48	0	0.933333333	6.226666667	2.16	0.68	1.373333333	0.773333333	0.333333333	0.253333333	0.613333333
14	inner	E	NE	0.441666667	0.201111111	0	0.375	2.087777778	0.041666667	1.042777778	0.12	0.160833333	0.842222222	0.245	8.753888889
14	outer	W	NW	3.453333333	4.08	0.48	1.04	3.28	0.6	0.493333333	0.346666667	0.746666667	0.52	0.586666667	2
15	inner	E	NE	0.04	0.06	0	0.08	0.16	0	0.253333333	0	0	0.48	0.4	14.74666667
15	outer	E	SE	2.410555556	0.548888889	0	0.948888889	0.416111111	0.161111111	0.213888889	0.22	0.28	1.602777778	4.275555556	0.898333333
16	outer	W	NW	1.786666667	3.76	0.04	0.4	2.826666667	1.88	0.28	0.866666667	1.026666667	1.106666667	0.52	0.146666667
17	outer	W	SW	0.44	0.706666667	0	0.72	2.626666667	0.826666667	0.36	0.613333333	0.32	1.44	1.466666667	0.733333333
18	outer	E	NE	0.826666667	2.226666667	0	0.266666667	0.666666667	0.28	0.28	0.08	0.16	0.84	9.733333333	1.22
20	outer	W	NW	0.84	2.693333333	0.06	0.96	1.946666667	0.826666667	0.746666667	0.48	0.586666667	2.706666667	2.146666667	1.08
